# Biocontrol of tobacco leaf mold using *Bacillus velezensis*: effects on volatile profiles and microbial community

**DOI:** 10.1128/spectrum.01131-26

**Published:** 2026-09-08

**Authors:** Daiyuan Zhang, Mo Zhang, Ge Zhang, Dian Zou, Dong Li, Huijing Gu, Xuetuan Wei, Kuo Huang, Changwen Ye

**Affiliations:** 1 Zhengzhou Tobacco Research Institute of China National Tobacco Corporation 154489, Zhengzhou, China; 2 National Key Laboratory of Agricultural Microbiology, College of Food Science and Technology, Huazhong Agricultural University 124443, Wuhan, China; Agroscope, Nyon, Switzerland

**Keywords:** *Bacillus velezensis*, tobacco leaves, antifungal effect, volatile compounds, microbial diversity

## Abstract

Mold contamination causes economic losses and poses risks to human and animal health. Tobacco leaves, as a high-value agricultural product, are particularly susceptible to mold contamination during post-harvest handling. In this study, the antifungal performance of *Bacillus velezensis* ZD-F13 was evaluated on tobacco leaves. In laboratory-scale assays, ZD-F13 treatment reduced the fungal colony count by 84% relative to the *Penicillium* group. Electronic-nose and gas chromatography-ion mobility spectrometry analyses showed that the volatile profile of the *Penicillium* + ZD-F13 group was closer to that of the normal leaves group than to that of the *Penicillium* group. To further evaluate its practical applicability, *B. velezensis* ZD-F13 was tested on 5 kg of tobacco leaves and reduced the fungal colony count by 50% relative to the untreated group. Microbial community analysis revealed changes in bacterial and fungal community composition, including an increase in the relative abundance of *Bacillus* from 0.09% to 48.1%. These findings support the potential application of *B. velezensis* ZD-F13 for controlling tobacco leaf mold and providing a basis for further development of postharvest biocontrol strategies.

**IMPORTANCE:**

Mold contamination of tobacco leaves causes substantial economic losses and can compromise product quality and safety during postharvest storage and processing. Effective control strategies remain limited, particularly those capable of suppressing mold without disturbing the natural microbial community or altering quality-related characteristics. This study showed that ZD-F13 treatment reduced fungal colony counts under controlled laboratory conditions and was associated with a lower fungal colony count in the 5-kg practical trial. ZD-F13 treatment also shifted the volatile profile toward that of healthy leaves without causing a statistically detectable loss of Shannon diversity under the tested conditions. These findings suggest that ZD-F13 has potential as a postharvest biocontrol agent for tobacco leaves.

## INTRODUCTION

Mold contamination causes substantial economic losses worldwide. It is estimated that 10%–23% of global crop production is lost annually as a result (1), and postharvest losses may reach up to 60% in regions with inadequate storage and transportation conditions (2). Meanwhile, mold contamination also poses significant public health risks. According to the World Health Organization, approximately 600 million people suffer from foodborne microbial contamination annually, with molds being a major contributing factor (https://www.who.int/news-room/fact-sheets/detail/food-safety). More importantly, many molds produce potent and stable mycotoxins, including aflatoxins and ochratoxins, which exhibit carcinogenic and teratogenic properties (3, 4). Consequently, mold contamination poses a serious challenge to food and agricultural safety. A variety of strategies have been employed to control mold contamination, including physical methods such as irradiation and modified-atmosphere storage, as well as chemical fungicides (5–7). Due to concerns over high energy consumption, the emergence of resistant strains, and chemical residues, there is an urgent need for environmentally sustainable alternatives (8).

*Bacillus* species have attracted considerable attention due to their strong antifungal activity and remarkable adaptability to various extreme environments (9–11). For instance, *Bacillus subtilis* has been proven to effectively control postharvest citrus decay caused by *Penicillium digitatum* (12). Similarly, *Bacillus amyloliquefaciens* has been shown to inhibit postharvest apple rot caused by *Aspergillus niger* and mold spoilage in tobacco leaves induced by *Penicillium chrysogenum* (13, 14). Among these agricultural commodities, tobacco leaves are of particular concern due to their high economic value and high susceptibility to mold contamination during postharvest processing (13, 15). Although the antifungal efficacy of *Bacillus* species has been demonstrated under laboratory conditions, their large-scale application in tobacco leaf preservation has not been systematically characterized. In addition to pursuing effective antifungal activity, it is equally important to evaluate the effect of biocontrol treatments on tobacco quality. Moreover, suppression of mold by biocontrol agents may affect microbial diversity and disrupt ecological functions, thereby limiting the practical application of biocontrol agents (16). Therefore, both volatile profiles and microbial community structure should be comprehensively evaluated. *B. velezensis* ZD-F13 was selected from our laboratory strain collection based on initial antifungal screening against the tobacco-derived isolate *P. chrysogenum* Z1. *P. chrysogenum* Z1 was prioritized because it had previously been isolated from mold-contaminated tobacco leaves and provided a reproducible challenge organism for the laboratory assay (13).

In this study, the antifungal activity of *Bacillus velezensis* ZD-F13 against mold contamination was evaluated on tobacco leaves under laboratory-scale conditions. The effects of *B. velezensis* ZD-F13 treatment on the volatile profile of tobacco leaves were examined using electronic-nose analysis and gas chromatography-ion mobility spectrometry (GC-IMS). To assess its practical applicability, a 5-kg tobacco leaf trial was subsequently conducted to determine the antifungal efficacy of *B. velezensis* ZD-F13 and its influence on the microbial community structure. Overall, this study provided valuable insights into the practical application potential of *B. velezensis* ZD-F13 for managing mold contamination.

## MATERIALS AND METHODS

### Laboratory-scale evaluation of the antifungal activity of *B. velezensis* ZD-F13

*B. velezensis* ZD-F13, previously preserved in our laboratory at Huazhong Agricultural University (Wuhan, China), was used to evaluate antifungal activity on air-cured tobacco leaves (Hubei, China) following a previously reported method (13). The strain was cultured in Luria-Bertani medium (10 g/L tryptone, 5 g/L yeast extract, and 10 g/L NaCl) at 37°C with shaking at 180 r/min for 12 h. The bacterial culture was then harvested by centrifugation at 5,500*g* for 5 min, washed twice with sterile water, and resuspended in sterile water to obtain a bacterial suspension of 10⁸ CFU/mL. *P. chrysogenum* Z1 was isolated from mold-contaminated tobacco leaves (Hubei, China) and preserved in our laboratory at Huazhong Agricultural University (Wuhan, China). *P. chrysogenum* was cultured on potato dextrose agar (PDA) plates at 28°C for 5 days. Spores were harvested by washing the colony surface with sterile water, and the suspension was filtered through sterile gauze to remove mycelial fragments. The spore concentration was determined using a hemocytometer and adjusted to 10⁵ CFU/mL with sterile water. The *in vitro* antifungal activity of ZD-F13 was evaluated using the agar well diffusion assay (13). Briefly, 100 μL of *P. chrysogenum* Z1 spore suspension (10⁵ CFU/mL) was evenly mixed with PDA medium. Oxford cups were placed on the plate to create uniform wells, and 100 μL of the ZD-F13 bacterial suspension (10⁸ CFU/mL) or the methanolic extract of the ZD-F13 fermentation supernatant was added to each well. The plates were incubated at 28°C for 7 days. Each treatment was performed in triplicate, and antifungal activity was evaluated based on the inhibition zones surrounding the agar wells. For preparation of the fermentation supernatant extract, ZD-F13 was cultured in 25 mL of fermentation medium containing 70 g/L soybean meal, 50 g/L glucose, 0.5 g/L K_2_HPO_4_·3H_2_O, 0.5 g/L MgSO_4_·7H_2_O, and 0.005 g/L MnSO_4_·H_2_O at 37°C and 180 r/min for 72 hours. The resulting fermentation supernatant (0.3 mL) was mixed with 1.2 mL of methanol and thoroughly vortexed. After centrifugation at 10,000*g* for 10 min, the extract was filtered through a 0.22-μm membrane and used for both the antifungal assay and high-performance liquid chromatography (HPLC) analysis. HPLC analysis of iturin A was performed using a LiChrospher C18 reverse-phase column (4.6 × 250 mm, 5 μm). The mobile phase consisted of 10 mmol/L ammonium acetate and acetonitrile (65:35, [vol/vol]), and UV detection was conducted at 210 nm.

For the tobacco-leaf assay, tobacco leaves (5 g) without visible infection were selected and sterilized with 1% sodium hypochlorite solution. The leaves were then rinsed with sterile water and air-dried under sterile conditions. For the laboratory-scale antifungal assay, the leaves were divided into two groups. Leaves inoculated with 1.5 mL of *P. chrysogenum* spore suspension and treated with sterile water instead of ZD-F13 bacterial suspension were defined as the *Penicillium* group. Leaves inoculated with 1.5 mL of *P. chrysogenum* spore suspension and treated with 1.5 mL of ZD-F13 bacterial suspension (10^8^ CFU/mL) were defined as the *Penicillium* + ZD-F13 group. All samples were then packaged and stored at 28°C for 7 d. Mold development on the leaf surfaces was visually evaluated. For fungal colony counts, tobacco leaf samples from each replicate were weighed and then homogenized in sterile water. The homogenates were serially diluted, and aliquots were spread onto PDA plates. After incubation at 28°C for 5 d, fungal colonies were counted and expressed as CFU/g tobacco leaves. Each group included three independent biological replicates. Data are presented as the mean ± standard deviation (SD). Differences between the two groups were analyzed using a two-tailed unpaired Student’s *t*-test, and *P* < 0.05 was considered statistically significant.

### E-nose analysis

For volatile-profile analysis, a normal leaves group was included as a healthy reference. This group consisted of uninoculated tobacco leaves treated with the same total volume of sterile water and incubated under the same conditions at 28°C for 7 days. No visible mold development was observed in the normal leaves group after incubation. The normal leaves, *Penicillium*, and *Penicillium* + ZD-F13 groups were used for electronic-nose analysis. The samples were analyzed using the PEN3 electronic nose system (AIRSENSE, Germany) following the method described by Ma et al. (17). Specifically, tobacco leaves (2 g) were placed into a 50 mL headspace vial. The sensor probe was inserted for 5 s, followed by an injection time of 100 s and a sensor cleaning time of 100 s. Both the carrier air flow rate and the sample flow rate were maintained at 400 mL/min. Sensor responses at three consecutive time points (59, 60, and 61 s) were averaged to obtain the final measurement for each sample. The sensor sensitivities were as follows: W1C for aromatic organic compounds, W5S for nitrogen oxides, W3C for ammonia and aromatic compounds, W6S for hydrides, W5C for alkenes, aromatics, and polar molecules, W1S for alkanes, W1W for sulfides, W2S for alcohols and some aromatic compounds, W2W for aromatic ingredients and organic sulfides, and W3S for alkanes and aliphatics. Each group included five independent biological replicate samples for electronic-nose analysis.

### GC-IMS analysis

The same three groups, including the normal leaves, *Penicillium*, and *Penicillium* + ZD-F13 groups, were used for GC-IMS analysis. The analytical procedure was adapted from Wang et al. (18) with minor modifications. Volatile compounds (VOCs) were analyzed using a GC-IMS system (FlavourSpec, Dortmund, Germany). A chopped sample (0.2 g) was weighed into a 20 mL glass vial. The vial was incubated at 80°C for 15 min with agitation at 500 rpm. Subsequently, a total of 500 µL headspace gas was withdrawn using a heated syringe (105°C) and injected in splitless mode. For GC separation, an MXT-WAX capillary column (15 m × 0.53 mm × 1.0 µm, Restek, USA) was used with the oven temperature maintained at 60°C. High-purity helium (≥99.999%) served as the carrier gas under the following programmed-flow conditions: 2 mL/min for 2 min, ramped to 10 mL/min within 8 min, further increased to 100 mL/min within 10 min, and held for 40 min. The inlet temperature was set at 80°C, and the total run time was 60 min. The IMS parameters were configured as follows: a tritium (^3^H) ionization source, drift tube length of 98 mm, and an electric field strength of 500 V/cm. The drift tube temperature was maintained at 45°C. High-purity nitrogen (≥99.999%) was used as the drift gas at 150 mL/min. All analyses were performed in positive ion mode. Each group included three independent biological replicates for GC-IMS analysis. Differences in VOC contents among groups were analyzed using one-way analysis of variance followed by Tukey’s test.

### Practical application of *B. velezensis* ZD-F13 on tobacco leaves under natural fungal contamination conditions

The method was based on that described in 2.1, with modifications for practical application under natural fungal contamination conditions. For each group, 5 kg of air-cured tobacco leaves (Hubei, China) were used. The tobacco leaves were divided into two groups. Leaves without bacterial treatment were defined as the untreated group, whereas leaves evenly sprayed with the ZD-F13 bacterial suspension at a concentration of 10⁸ CFU/mL were defined as the ZD-F13-treated group. After treatment, the leaves were arranged on bamboo curing racks in an air-curing room under natural ventilation. During the 14-day trial, the ambient temperature ranged from approximately 28°C to 32°C, and the relative humidity ranged from approximately 70% to 85%. After 14 days, mold development on the leaves was visually evaluated. Fungal colony counts were determined using the same culture-based dilution plating method described in section Laboratory-scale evaluation of the antifungal activity of *B. velezensis* ZD-F13. Briefly, tobacco leaf samples from each replicate were weighed and then homogenized in sterile water. The homogenates were serially diluted 10-fold, and aliquots of appropriate dilutions were spread onto PDA plates. After incubation at 28°C for 5 days, fungal colonies were counted and expressed as CFU/g tobacco leaves. Each group included three independent biological replicates. Data are presented as the mean ± SD. Differences between the two groups were analyzed using a two-tailed unpaired Student’s *t*-test, and *P* < 0.05 was considered statistically significant.

### Microbial community analysis

For microbial community analysis, tobacco leaf samples were collected from the untreated and ZD-F13-treated groups in the 5-kg practical trial. Each group included four independent biological replicates. For each biological replicate, tobacco leaves were randomly collected from different positions within the corresponding treatment and pooled as one composite sample. Genomic DNA was extracted using the E.Z.N.A. Plant DNA Kit (Omega Bio-tek, Norcross, GA, USA) following the manufacturer’s instructions. The V3–V4 region of the bacterial 16S rRNA gene and the fungal ITS region were amplified using primers 338F/806R and ITS1F/ITS2R, respectively, as previously described (19). Paired-end reads were merged using FLASH (v1.2.7), and low-quality sequences were filtered using fastp (v0.19.6) (20). Operational taxonomic units (OTUs) were used for downstream microbial community analysis. Taxonomic assignment of OTUs was performed against the SILVA v138.1 and UNITE v9.0 databases for bacteria and fungi, respectively (21). Alpha diversity was calculated using the Shannon index and Simpson dominance index, and differences in alpha diversity between the untreated and ZD-F13-treated groups were analyzed using the Wilcoxon rank-sum test. Beta diversity was assessed by principal coordinate analysis (PCoA) based on Bray-Curtis distances, and differences between groups were tested using analysis of similarities (ANOSIM) with 999 permutations on the Majorbio Cloud Platform (22). Significantly enriched taxa (biomarkers) were identified using linear discriminant analysis effect size (LEfSe) with an LDA score >3 and Kruskal-Wallis test, *P* < 0.05 (23).

## RESULTS AND DISCUSSION

### Laboratory-scale assessment of the antifungal activity of *B. velezensis* ZD-F13

**Fig 1:**
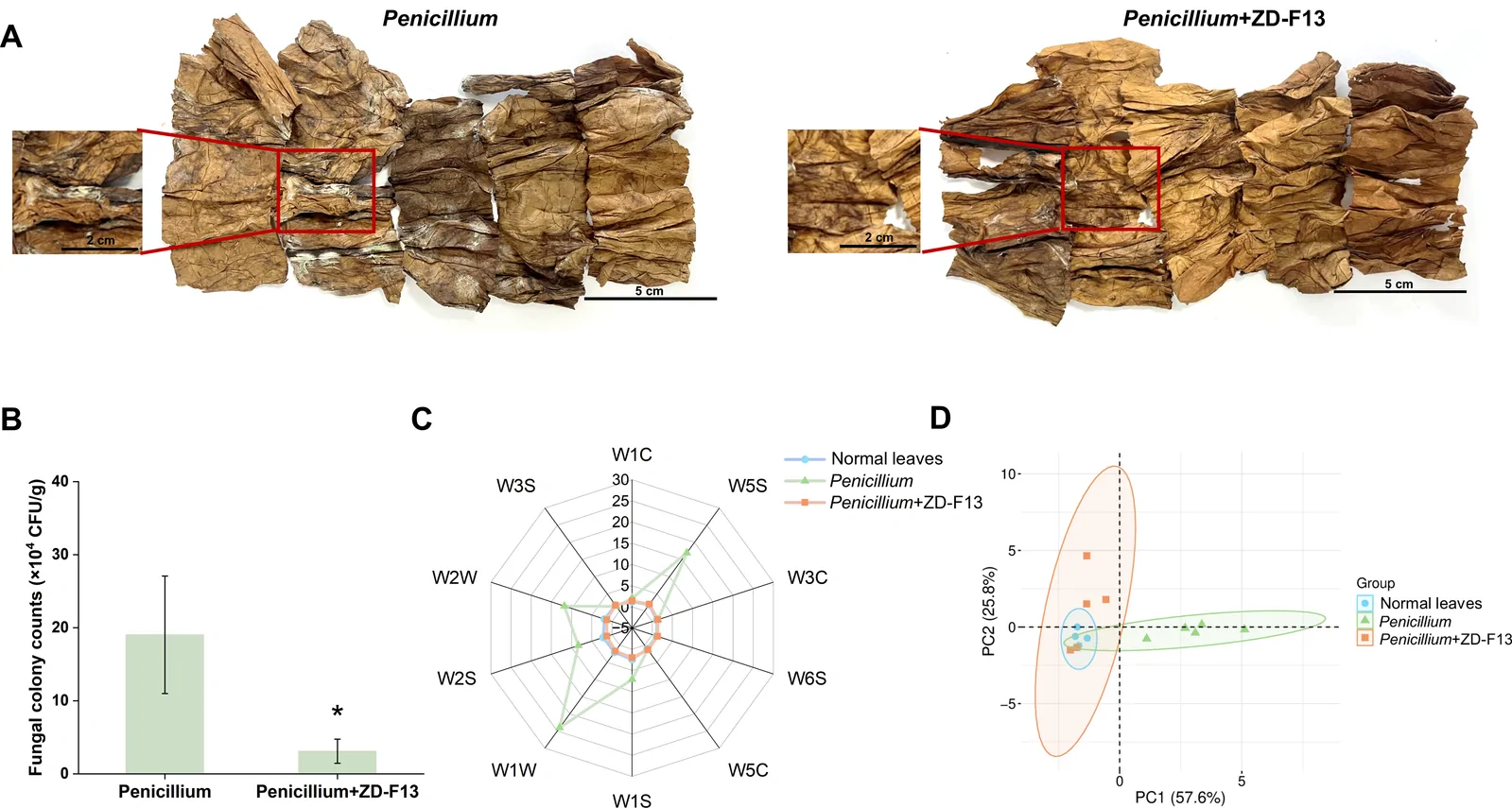
Laboratory-scale assessment of the antifungal efficacy of *B. velezensis* ZD-F13 on tobacco leaves. (**A**) Visual antifungal effect of the ZD-F13 suspension on tobacco leaves. (**B**) Fungal colony counts. (**C**) Radar plot of volatile profiles of tobacco leaves from different groups based on electronic-nose analysis. (**D**) PCA score plot based on electronic-nose analysis. Scale bars in the whole-view and close-up images represent 5 cm and 2 cm, respectively. Data in panel **B** are presented as the mean ± SD of three biological replicates. The asterisk represents the significance of the difference compared with the *Penicillium* group. *, *P* < 0.05.

To assess the direct antifungal activity of ZD-F13, agar well diffusion assays were performed. Both the ZD-F13 bacterial suspension and the methanolic extract of its fermentation supernatant inhibited the growth of *P. chrysogenum* Z1, whereas methanol showed no comparable inhibition (Fig. S1A and B), suggesting the involvement of secreted metabolites. HPLC analysis revealed peaks with retention times consistent with those of the iturin A standard (Fig. S1C). Because iturin A is a cyclic lipopeptide produced by *Bacillus* species and has documented antifungal activity (8), these findings suggest that ZD-F13 may exert its antifungal activity through the secretion of iturin A or related extracellular lipopeptides. However, further studies are needed to elucidate the specific antifungal mechanisms involved. Tobacco leaves, as a high-value agricultural product, are susceptible to mold contamination during post-harvest handling (24, 25). The early air-curing period represents an important stage for mold development because of the relatively high moisture content of the leaves. *Penicillium* species are frequently isolated from mold-contaminated tobacco leaves, and their growth has been associated with uneven surface coloration and the formation of pungent off-flavors (13, 26, 27). Therefore, the ability of the *B. velezensis* ZD-F13 bacterial suspension to control *P. chrysogenum* contamination on air-cured tobacco leaves was evaluated under laboratory conditions. Compared with the *Penicillium* group inoculated with *P. chrysogenum* spore suspension only, treatment with the *B. velezensis* ZD-F13 suspension at 10⁸ CFU/mL reduced visible mold development on the tobacco leaf surface (Fig. 1A). Fungal colony enumeration showed that the fungal population in the *Penicillium* + ZD-F13 group was 84% lower than that in the *Penicillium* group (*P* = 0.028; Fig. 1B). These results indicate that *B. velezensis* ZD-F13 suppressed *P. chrysogenum* growth on tobacco leaves under the tested laboratory conditions.

The effects of the bacterial suspension treatment on the volatile-response profile of tobacco leaves were further investigated using an electronic nose system. Compared with the normal leaves group, the *Penicillium* group showed higher responses in several sensor channels, including W5S (nitrogen oxides), W1S (methane-related compounds), W1W (sulfides), W2S (ethanol), and W2W (aromatic compounds and organic sulfides; Fig. 1C). These changes involved multiple sensor categories rather than being confined to a single type of sensor response, indicating a broad alteration in the electronic-nose response profile associated with *P. chrysogenum* growth. The higher responses of sulfide-sensitive sensors in the *Penicillium* group may be associated with the development of fishy or putrid off-odors (28, 29). In contrast, the *Penicillium* + ZD-F13 group showed lower responses than the *Penicillium* group in these sensor channels and an overall response pattern closer to that of the normal leaves group. This pattern was consistent with the lower fungal population in the *Penicillium* + ZD-F13 group, suggesting that reduced *P. chrysogenum* growth was accompanied by fewer mold-associated changes in the overall volatile-response profile. To further visualize the overall differences among the electronic-nose response profiles, principal component analysis (PCA) was performed. As shown in Fig. 1D, PC1 and PC2 accounted for 57.6% and 25.8% of the total variance, respectively, together explaining 83.4% of the variability in the data set. The normal leaves group and the *Penicillium* + ZD-F13 group occupied adjacent regions and showed partial overlap in the PCA score plot, indicating similar overall electronic-nose response patterns between the two groups. In contrast, the *Penicillium* group was separated from the normal leaves group primarily along the PC1 axis, showing that *P. chrysogenum* growth altered the overall volatile-response pattern detected by the electronic nose. The closer position of the *Penicillium* + ZD-F13 group to the normal leaves group suggests that ZD-F13 treatment reduced these mold-associated changes.

### Characterization and relative abundance of volatile organic compounds

**Fig 2:**
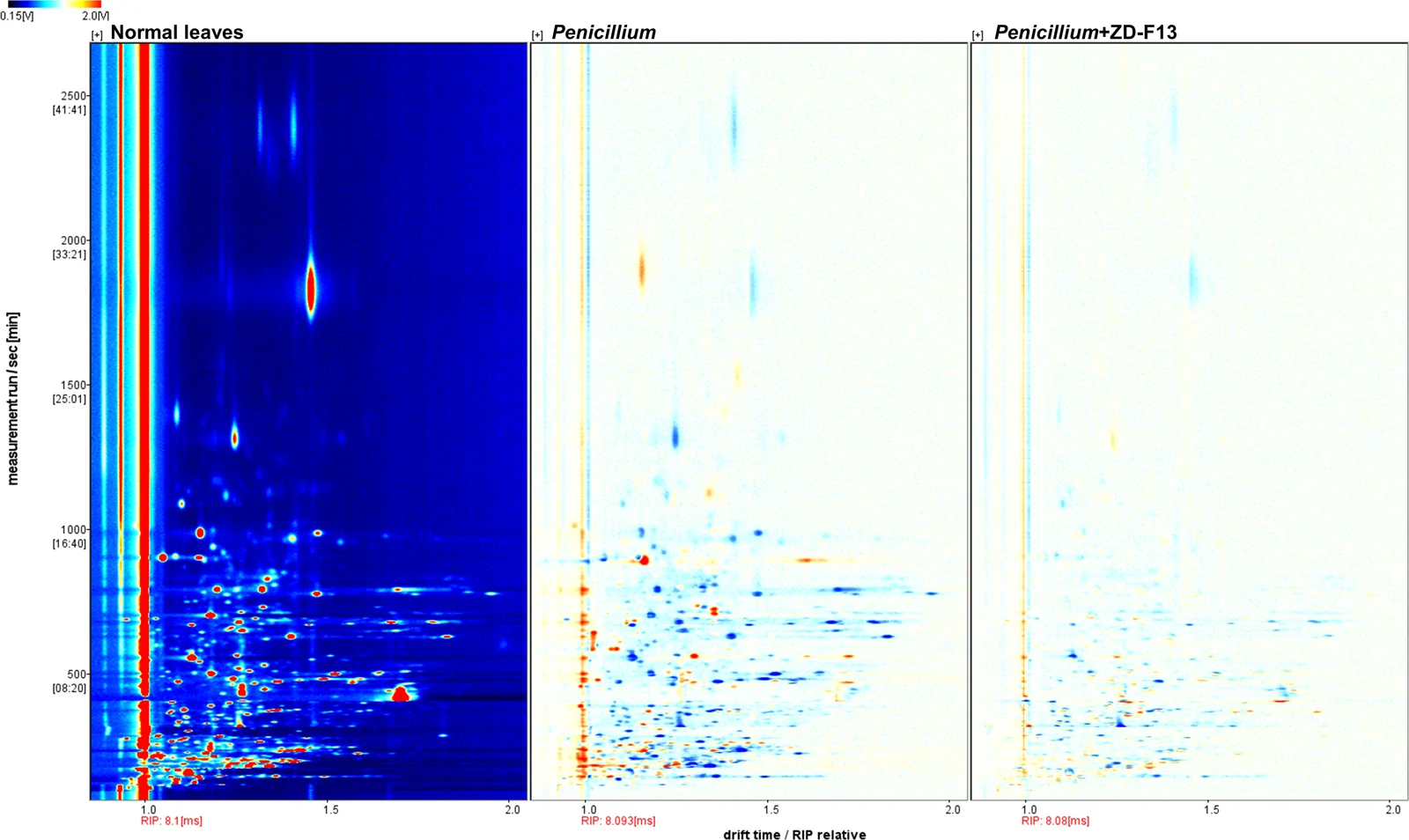
Differential analysis of VOCs among groups based on GC-IMS. Red and blue regions represent increases and decreases in VOCs relative to the normal leaves group, respectively, with color intensity indicating the magnitude of change.

GC-IMS was employed to further characterize differences in the VOC profiles among the groups. The VOC signals were mainly distributed within a retention time range of 100–2,500 s, and certain compounds produced multiple signals (monomers, dimers, or even trimers), depending on their concentration and chemical properties (30). Using the spectrum of the normal leaves group as the reference, the spectra of the *Penicillium* group and the *Penicillium* + ZD-F13 group were converted into two-dimensional differential contour plots by subtracting the signals of the normal leaves group. As shown in Fig. 2, white regions indicate comparable signal intensities between a treatment group and the normal leaves group, whereas blue regions indicate lower signal intensities, with darker shades representing greater decreases (31). In contrast, red regions indicate higher signal intensities, with darker red representing a greater increase. Compared with the normal leaves group, the *Penicillium* group exhibited more extensive red and blue regions, whereas fewer differential regions were observed in the *Penicillium* + ZD-F13 group. This pattern indicates that *P. chrysogenum* growth altered the overall VOC profile and that these changes were reduced following ZD-F13 treatment.

To identify the VOC signals contributing to the differences among the groups, VOCal software combined with the GC-IMS database was used to assign compounds based on their drift times and retention indices. A total of 154 VOC signals, including monomeric, dimeric, and trimeric signals, were detected. As summarized in Table S1, the detected signals comprised 35 aldehydes, 24 alcohols, 17 ketones, 6 esters, 3 acids, 7 terpenes, 12 nitrogen-containing compounds, 5 sulfur compounds, 1 oxygenated compound, 2 furans, 1 aromatic compound, and 41 additional features that could not be assigned due to the lack of matching entries in the GC-IMS library. As shown in Fig. S2, regions a, b, and d contained multiple signals with lower intensities in the *Penicillium* group than in the normal leaves group, whereas region c contained 16 elevated signals distributed across four chemical classes rather than being confined to a single class. These included nitrogen compounds (2,3-dimethylpyrazine, 2,5-dimethylpyrazine, 2-methylpyrazine-M, 2-ethylpyridine, 1H-pyrrole, and 2-acetyl-2-thiazoline), ketones (4-heptanone, 2-pentanone, 2-heptanone, 5-nonanone, and 2-butanone), aldehydes (heptaldehyde, 2-methylpropanal, and (E)-4-decenal), and alcohols (1-octen-3-ol-D and 1-octen-3-ol-M). These changes may have arisen through both the transformation of endogenous tobacco constituents during *P. chrysogenum* growth and the production of volatile metabolites by *P. chrysogenum* itself. In particular, 1-octen-3-ol and 2-pentanone have previously been detected among volatile metabolites emitted during *P. chrysogenum* growth, suggesting that their increased abundance in the *Penicillium* group may partly reflect direct fungal production rather than changes in the baseline tobacco aroma (32). Future studies involving separate characterization of the *P. chrysogenum* volatilome could further clarify the fungal origin of these VOCs.

### Multivariate statistical analysis of volatile organic compounds

**Fig 3:**
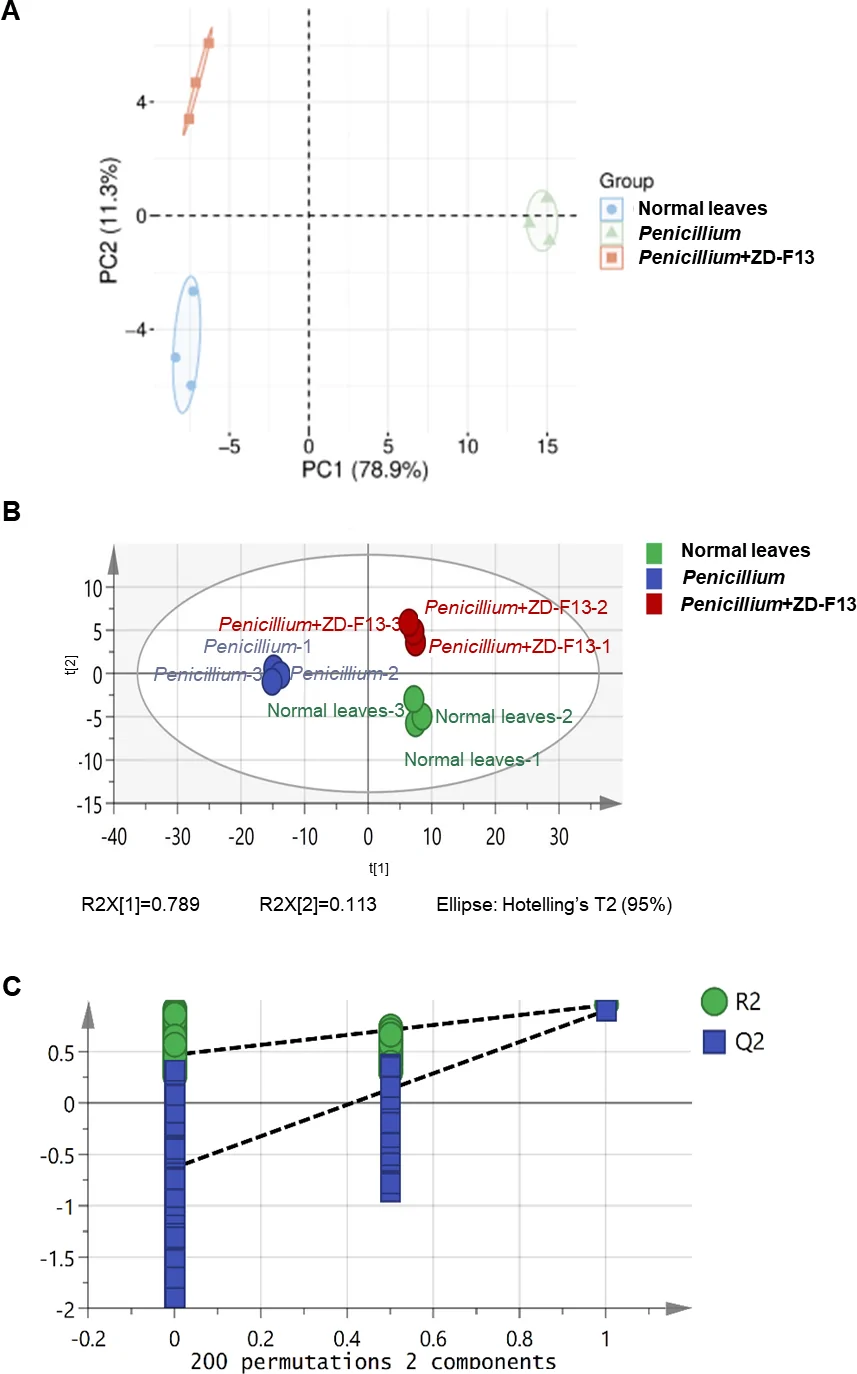
Multivariate statistical analysis of volatile compounds in tobacco leaves from different groups. (**A**) PCA score plot. (**B**) OPLS-DA score plot. (**C**) A total of 200-iteration permutation test for model validation.

PCA was used to evaluate the overall variation in the VOC profiles among the groups (33). As shown in Fig. 3A, PC1 and PC2 accounted for 78.9% and 11.3% of the total variance, respectively, together explaining 90.2% of the variance in the data set. The *Penicillium* group was separated from the normal leaves group primarily along PC1, whereas the *Penicillium* + ZD-F13 group was positioned closer to the normal leaves group. This distribution was consistent with the reduced VOC changes associated with *P. chrysogenum* growth in the *Penicillium* + ZD-F13 group. To further evaluate supervised separation among the groups, an orthogonal partial least squares discriminant analysis (OPLS-DA) model was constructed. As shown in Fig. 3B, the three groups formed separate clusters within the 95% Hotelling’s *T*² ellipse, and the model yielded *R*²*X* = 0.902, *R*²*Y* = 0.970, and *Q*² =0.942. A 200-iteration permutation test produced a *Q*² intercept of −0.612 (Fig. 3C). The negative *Q*² intercept supported the absence of overfitting in the OPLS-DA model. Variable importance in projection (VIP) scores were used to identify compounds contributing to group separation. A VIP >1 is commonly used to indicate a relatively high contribution to the model (34). As shown in Fig. S3, 38 VOC signals had VIP >1 and were therefore identified as variables contributing to the differentiation among the groups.

### Practical evaluation of *Bacillus velezensis* ZD-F13 for controlling natural mold contamination in tobacco leaves

**Fig 4:**
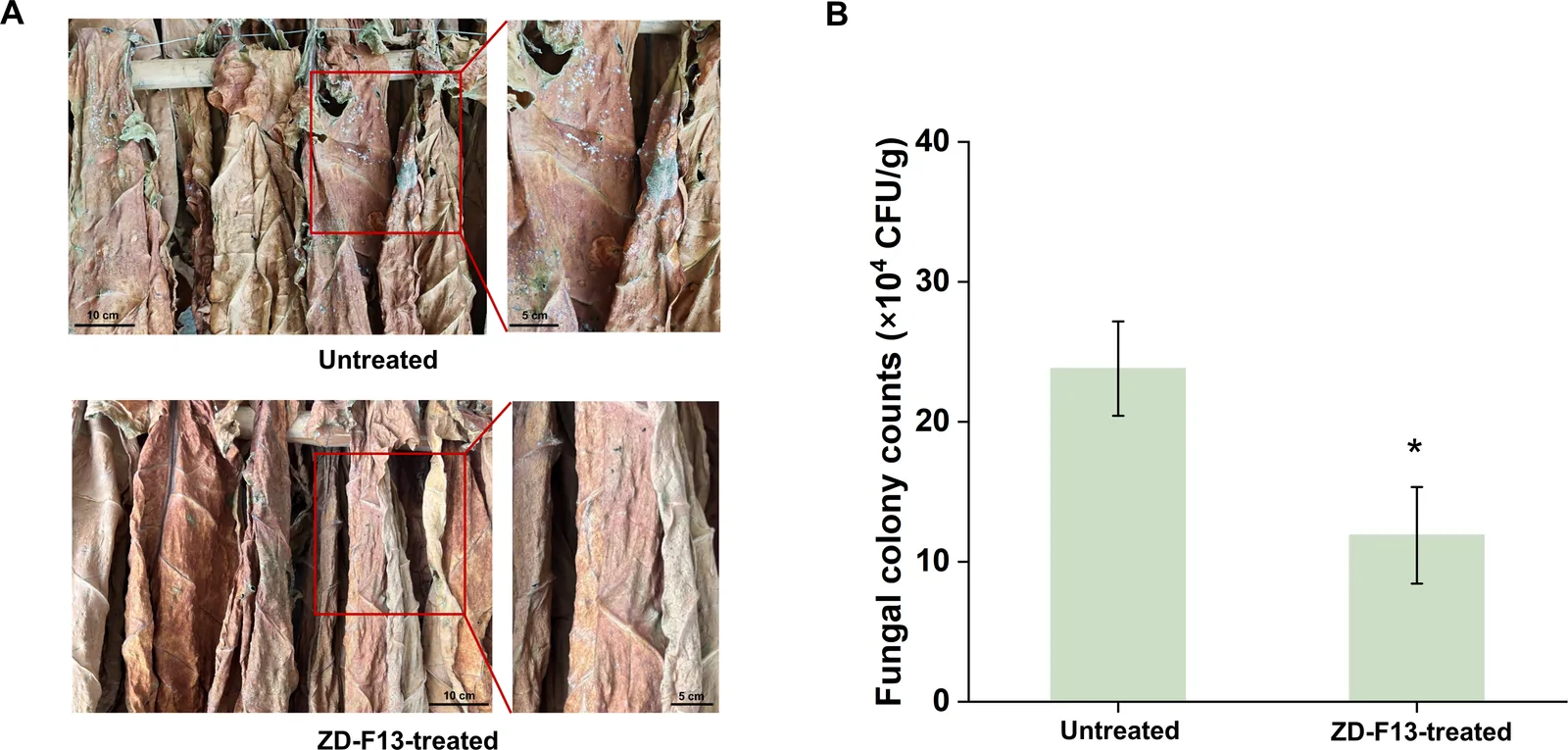
Practical evaluation of the antifungal efficacy of *B. velezensis* ZD-F13 on tobacco leaves. (**A**) Visual antifungal effect of the ZD-F13 suspension on tobacco leaves. (**B**) Fungal colony counts. Scale bars in the whole-view and close-up images represent 10 cm and 5 cm, respectively. Data in panel **B** are presented as the mean ± SD of three biological replicates. The asterisk represents the significance of the difference compared with the untreated group. *, *P* < 0.05.

A 5-kg practical trial was conducted using air-cured tobacco leaves to evaluate whether the reduction in mold contamination observed in the laboratory assay could also be detected under practical post-harvest conditions with naturally occurring mold. As shown in Fig. 4A, the ZD-F13-treated group showed less visible mold development on the tobacco leaf surface than the untreated group. Fungal colony enumeration showed that the fungal colony count in the ZD-F13-treated group was 50% lower than that in the untreated group (*P* = 0.013) after treatment with 10⁸ CFU/mL of the ZD-F13 bacterial suspension (Fig. 4B). These results indicated that *B. velezensis* ZD-F13 reduced fungal contamination under the tested 5-kg post-harvest conditions. Unlike the laboratory assay based on artificial inoculation with *P. chrysogenum*, the practical trial involved naturally occurring mold contamination within a complex microbial community. Therefore, the reduction in fungal contamination observed in this trial cannot be attributed solely to the suppression of a single fungal species and may also be associated with changes in microbial community composition or interactions among resident microorganisms (35–37). This distinction motivated the subsequent characterization of the bacterial and fungal communities in the untreated and ZD-F13-treated groups.

### Effects of ZD-F13 treatment on bacterial community structure and diversity

**Fig 5:**
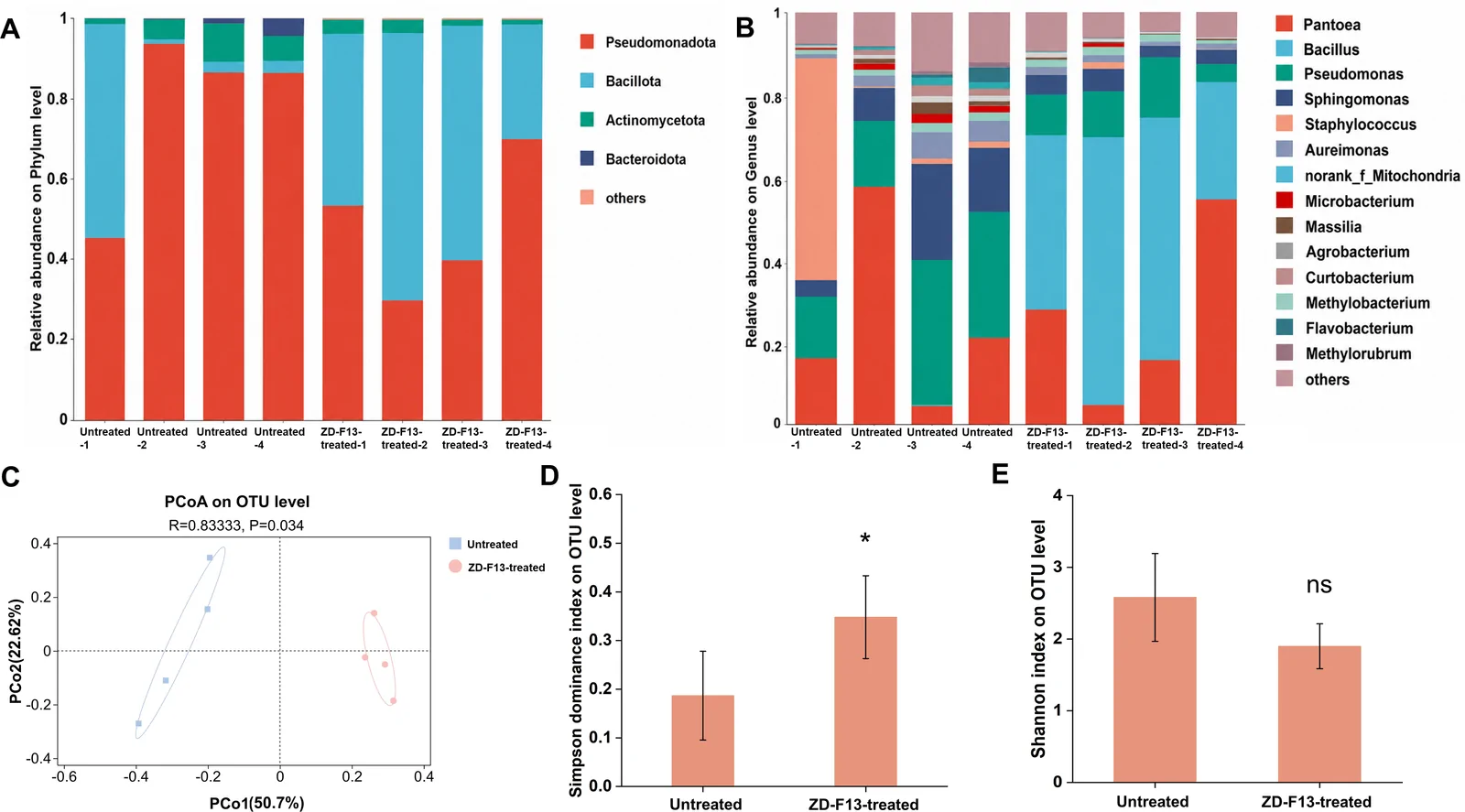
Effects of ZD-F13 treatment on bacterial community structure and diversity. (**A**) Taxonomic composition at the phylum level. (**B**) Taxonomic composition at the genus level. (**C**) β-diversity analysis based on Bray-Curtis dissimilarity using ANOSIM with 999 permutations. (**D**) Simpson dominance index. (**E**) Shannon index. Data in panels **D** and **E** are presented as the mean ± SD of four biological replicates. The asterisk represents the significance of the difference compared with the untreated group. *, *P* < 0.05, and ns, not significant.

16S rRNA gene sequencing was performed to assess the bacterial community structure in post-harvest tobacco leaves. At the phylum level, the relative abundance of Pseudomonadota was lower, whereas that of Bacillota was higher, in the ZD-F13-treated group than in the untreated group (Fig. 5A). At the genus level, the relative abundance of *Bacillus* increased from 0.09% in the untreated group to 48.1% in the ZD-F13-treated group and became the dominant bacterial genus (Fig. 5B). Because amplicon sequencing provides relative-abundance data, the reduced proportions of other taxa did not necessarily indicate decreases in their absolute population sizes. Wilcoxon rank-sum tests were used to compare genus-level relative abundances between the two groups (Fig. S4A). *Bacillus*, *Glutamicibacter*, and *Cellulomonas* were more abundant in the ZD-F13-treated group, whereas *Pseudomonas*, *Curtobacterium*, and *Flavobacterium* were more abundant in the untreated group. LEfSe analysis identified *Bacillus* as enriched in the ZD-F13-treated group (Fig. S4B). This enrichment was consistent with ZD-F13 application. However, genus-level 16S rRNA gene sequencing could not distinguish the applied strain from indigenous *Bacillus* populations. Strain-specific quantification would be required to confirm the persistence of ZD-F13. Members of the genus *Bacillus* can suppress fungal growth through mechanisms such as antifungal metabolite production and competition for nutrients and space (11, 38). Previous studies have reported the application of *Bacillus* in tobacco fermentation and its potential to reduce tobacco-specific nitrosamines (39, 40). However, tobacco-specific nitrosamines were not quantified in the present study. The observed enrichment of *Bacillus* could not be interpreted as evidence of reduced nitrosamine levels or improved chemical safety.

β-Diversity based on Bray-Curtis dissimilarity was assessed using PCoA. The untreated and ZD-F13-treated groups occupied separate regions of the PCoA plot, and ANOSIM indicated a difference in bacterial community composition between the two groups (*R* = 0.83, *P* = 0.034; Fig. 5C). The first two principal coordinates, PCo1 and PCo2, explained 50.7% and 22.62% of the total variation, respectively, together accounting for 73.32%. The α-diversity analysis revealed that the Simpson dominance index was higher in the ZD-F13-treated group than in the untreated group (*P* = 0.035; Fig. 5D). This increase reflected greater *Bacillus* dominance rather than greater community evenness (41). In contrast, the Shannon index did not differ significantly between the untreated and ZD-F13-treated groups (*P* = 0.094; Fig. 5E), indicating no statistically detectable change in Shannon diversity (42). Together, these results indicate that ZD-F13 treatment altered bacterial community composition and increased *Bacillus* dominance.

### Effects of ZD-F13 treatment on fungal community structure and diversity

**Fig 6:**
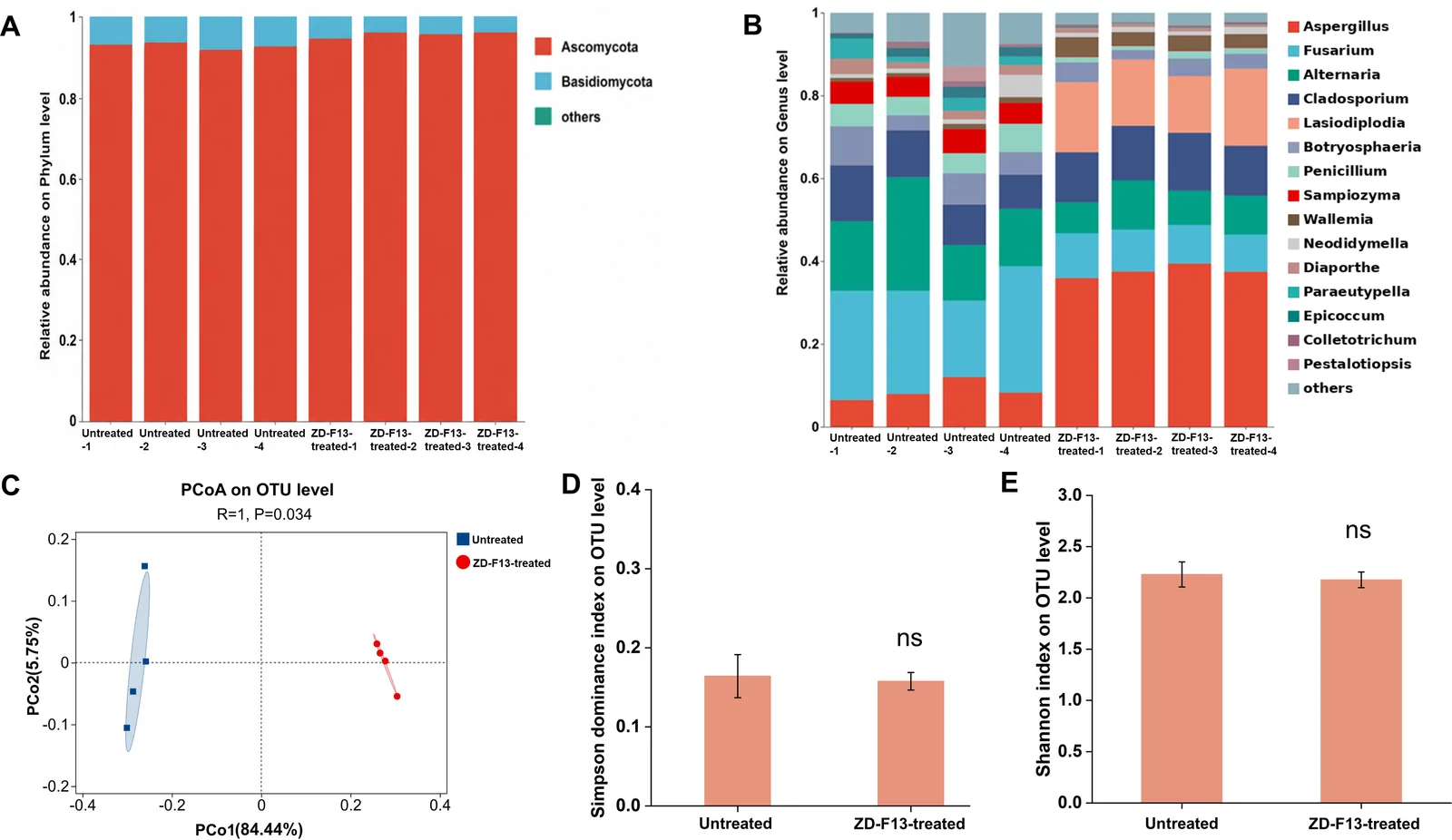
Effects of ZD-F13 treatment on fungal community structure and diversity. (**A**) Taxonomic composition at the phylum level. (**B**) Taxonomic composition at the genus level. (**C**) β-diversity analysis based on Bray-Curtis dissimilarity using ANOSIM with 999 permutations. (**D**) Simpson dominance index. (**E**) Shannon index. Data in panels **D** and **E** are presented as the mean ± SD of four biological replicates. ns indicates no significant difference compared with the untreated group.

ITS amplicon sequencing was used to characterize the fungal community structure of post-harvest tobacco leaves. At the phylum level, the relative abundance of Ascomycota increased from 92.82% in the untreated group to 95.58% in the ZD-F13-treated group, whereas that of Basidiomycota decreased from 7.16% to 4.41% (Fig. 6A). At the genus level, the relative abundance of *Fusarium* decreased from 25.08% in the untreated group to 9.79% in the ZD-F13-treated group. The relative abundance of *Penicillium*, the genus used in the laboratory-scale inoculation assay, decreased from 5.39% to 1.38%. In contrast, the relative abundance of *Aspergillus* increased from 8.69% to 37.61% and became the dominant fungal genus in the ZD-F13-treated group (Fig. 6B). Because amplicon sequencing provides relative-abundance data, the increase in *Aspergillus* does not necessarily indicate a corresponding increase in its absolute abundance. Wilcoxon rank-sum tests were used to compare genus-level relative abundances between the two groups (Fig. S5A). *Aspergillus* and *Lasiodiplodia* showed higher relative abundances in the ZD-F13-treated group, whereas several spoilage-associated genera, including *Fusarium*, *Alternaria*, *Penicillium*, *Sampaiozyma*, *Diaporthe*, *Paraeutypella*, and *Epicoccum*, were more abundant in the untreated group. LEfSe analysis identified g_*Fusarium* as a discriminatory taxon in the untreated group and o_*Eurotiales* as a discriminatory taxon in the ZD-F13-treated group (Fig. S5B). Some *Fusarium* species can produce mycotoxins and cause tobacco diseases under field conditions (43). Accordingly, the lower relative abundances of *Fusarium* and several other spoilage-associated genera in the ZD-F13-treated group were consistent with the observed reduction in visible mold severity. However, the increased relative abundance of *Aspergillus* warrants attention because some species are mycotoxigenic (44). Because genus-level ITS sequencing could not identify toxigenic species and mycotoxins were not quantified, the present study cannot determine whether ZD-F13 treatment altered mycotoxin-related risk. Species-specific absolute quantification and targeted mycotoxin analysis are therefore needed.

The β-diversity analysis based on Bray-Curtis dissimilarity was evaluated using PCoA. The untreated and ZD-F13-treated groups occupied separate regions of the PCoA plot, and ANOSIM indicated a difference in fungal community composition (*R* = 1, *P* = 0.034; Fig. 6C). The first two principal coordinates explained 84.44% (PCo1) and 5.75% (PCo2) of the total variation, together accounting for 90.19%. The α-diversity analysis showed no statistically detectable differences in either the Simpson dominance index (*P* = 0.652; Fig. 6D) or the Shannon index (*P* = 0.481; Fig. 6E) between the two groups. Together, these results indicate that ZD-F13 treatment was associated with altered fungal community composition, whereas no statistically detectable change in fungal α-diversity was observed.

### Conclusion

This study demonstrated that treatment with the *B. velezensis* ZD-F13 suspension reduced the fungal colony count on tobacco leaves by 84% in laboratory-scale assays. Electronic-nose and GC-IMS analyses showed that the volatile profile of the *Penicillium* + ZD-F13 group was closer to that of the normal leaves group, as indicated by their adjacent and partially overlapping positions in the PCA score plot, while differing from that of the *Penicillium* group. Additionally, 154 volatile signals were detected, of which 38 showed VIP > 1, providing information for understanding mold-associated volatile alterations and identifying potential indicators for quality monitoring. In a practical evaluation using 5 kg of tobacco leaves, *B. velezensis* ZD-F13 treatment reduced the fungal colony count by 50% relative to the untreated group. Microbial community analysis further indicated that the relative abundance of *Bacillus* increased from 0.09% to 48.1%, although genus-level 16S rRNA gene sequencing could not confirm the persistence of the applied strain. Overall, these findings support the potential application of ZD-F13 for controlling tobacco leaf mold, while highlighting the need for strain-specific quantification.

## Data Availability

The raw bacterial 16S rRNA gene and fungal ITS amplicon sequencing reads generated in this study have been deposited in the NCBI Sequence Read Archive under BioProject accession number PRJNA1506693. The GC-IMS data are publicly available in the Zenodo repository at https://doi.org/10.5281/zenodo.21783709.

## SUPPLEMENTAL MATERIAL

Table S1; Figures S1 to S5 spectrum.01131-26-s0001.docx Table S1: VOCs detected by GC-IMS in different groups. Figure S1: Antifungal activity and extracellular metabolite analysis of *B. velezensis* ZD-F13. Figure S2: GC-IMS fingerprint profiles of VOCs across different groups. Figure S3: VOCs with VIP > 1 identified by OPLS-DA. Figure S4: Differential abundance analyses of bacterial taxa between the groups. Figure S5: Differential abundance analyses of fungal taxa between the groups.

ASM does not own the copyrights to Supplemental Material that may be linked to, or accessed through, an article. The authors have granted ASM a non-exclusive, world-wide license to publish the Supplemental Material files. Please contact the corresponding author directly for reuse.
