# Supplementary material for: Biocontrol of tobacco leaf mold using Bacillus velezensis: effects on volatile profiles and microbial community
Source: Microbiol Spectr. 2026 Sep 8;14(10):e01131-26. doi: 10.1128/spectrum.01131-26 (PMC13689419; doi:10.1128/spectrum.01131-26)
Supplement: Supplementary file 1 [file spectrum.01131-26-s0001.docx]

**Supplementary material**

**Biocontrol of tobacco leaf mold using *Bacillus velezensis*: Effects on volatile profiles and microbial community**

**Table S1.** VOCs detected by GC-IMS in different groups.

| NO. | Volatile compounds | CAS# | Formula | Retention  Index (RI) | Retention time  (Rt [s]) | Relative content (%) Normal leaves | Relative content (%) *Penicillium* | Relative content (%) *Penicillium*+ZD-F13 | Flavor attribute |
| --- | --- | --- | --- | --- | --- | --- | --- | --- | --- |
| Aldehydes | |  |  |  |  |  |  |  |  |
| 1 | Acetaldehyde | C75070 | C_2_H_4_O | 671.2 | 108.802 | 0.31 ± 0.01^b^ | 0.46 ± 0.02^a^ | 0.31 ± 0.01^b^ | green, slight fruity |
| 2 | Propanal | C123386 | C_3_H_6_O | 803.6 | 144.847 | 1.13 ± 0.10^c^ | 1.92 ± 0.02^a^ | 1.56 ± 0.04^b^ | pungent, green grassy |
| 3 | 2-Methyl propanal | C78842 | C_4_H_8_O | 820.1 | 150.127 | 0.10 ± 0.00^c^ | 0.35 ± 0.03^a^ | 0.17 ± 0.01^b^ | banana, melon, slightly nutty |
| 4 | Butanal | C123728 | C_4_H_8_O | 896.9 | 177.236 | 1.14 ± 0.09^a^ | 1.27 ± 0.09^a^ | 1.07 ± 0.08^a^ | pungent, fruity, green leaf |
| 5 | 3-Methyl butanal | C590863 | C_5_H_10_O | 938.6 | 197.301 | 2.08 ± 0.15^b^ | 2.60 ± 0.02^a^ | 1.92 ± 0.10^b^ | chocolate, fat |
| 6 | n-Pentanal-D | C110623 | C_5_H_10_O | 998.9 | 236.043 | 3.36 ± 0.13^b^ | 1.60 ± 0.02^c^ | 3.89 ± 0.19^a^ | green grassy, faint banana, pungent |
| 7 | n-Pentanal-M | C110623 | C_5_H_10_O | 1000.2 | 237.022 | 0.60 ± 0.04^b^ | 0.75 ± 0.01^a^ | 0.66 ± 0.04^b^ | green grassy, faint banana, pungent |
| 8 | (E)-2-Butenal-M | C123739 | C_4_H_6_O | 1059.9 | 284.585 | 1.49 ± 0.03^a^ | 1.62 ± 0.13^a^ | 1.54 ± 0.02^a^ |  |
| 9 | (E)-2-Butenal-D | C123739 | C_4_H_6_O | 1062.2 | 286.61 | 1.79 ± 0.14^a^ | 0.69 ± 0.13^b^ | 1.52 ± 0.10^a^ |  |
| 10 | 1-hexanal | C66251 | C_6_H_12_O | 1094.6 | 317.632 | 1.05 ± 0.25^a^ | 0.59 ± 0.23^a^ | 0.40 ± 0.48^a^ | fresh, green, fat, fruity |
| 11 | (Z)-2-pentenal-D | C1576869 | C_5_H_8_O | 1108.5 | 333.815 | 0.19 ± 0.04^a^ | 0.00 ± 0.00^b^ | 0.14 ± 0.01^a^ |  |
| 12 | (Z)-2-pentenal-M | C1576869 | C_5_H_8_O | 1108.8 | 334.203 | 0.57 ± 0.05^a^ | 0.51 ± 0.03^a^ | 0.54 ± 0.03^a^ |  |
| 13 | 2-Methyl-2-butenal | C1115113 | C_5_H_8_O | 1119.6 | 347.435 | 0.28 ± 0.03^a^ | 0.21 ± 0.02^b^ | 0.24 ± 0.02^ab^ |  |
| 14 | (E)-2-Pentenal-M | C1576870 | C_5_H_8_O | 1138.5 | 371.711 | 1.12 ± 0.01^b^ | 1.31 ± 0.06^a^ | 1.21 ± 0.02^b^ | potato, peas |
| 15 | (E)-2-Pentenal-D | C1576870 | C_5_H_8_O | 1141.2 | 375.245 | 0.79 ± 0.10^a^ | 0.75 ± 0.04^a^ | 0.92 ± 0.06^a^ | potato, peas |
| 16 | Heptaldehyde | C111717 | C_7_H_14_O | 1193.2 | 449.065 | 0.57 ± 0.02^b^ | 0.98 ± 0.08^a^ | 0.52 ± 0.00^b^ | fresh, aldehyde, fatty, green herbs, wine, fruity |
| 17 | 3-Methyl-2-butenal-M | C107868 | C_5_H_8_O | 1208.8 | 470.774 | 0.87 ± 0.01^a^ | 0.56 ± 0.04^b^ | 0.85 ± 0.03^a^ | fruity |
| 18 | 3-Methyl-2-butenal-D | C107868 | C_5_H_8_O | 1210 | 472.525 | 1.84 ± 0.06^a^ | 0.27 ± 0.04^c^ | 1.24 ± 0.18^b^ | fruity |
| 19 | (Z)-2-Hexenal | R218138 | C_6_H_10_O | 1219.8 | 486.732 | 0.08 ± 0.01^c^ | 0.12 ± 0.01^a^ | 0.10 ± 0.00^b^ |  |
| 20 | (E)-2-hexenal-D | C6728263 | C_6_H_10_O | 1229.9 | 501.795 | 1.72 ± 0.10^a^ | 0.26 ± 0.03^b^ | 1.78 ± 0.03^a^ | green, banana, fat |
| 21 | (E)-2-hexenal-M | C6728263 | C_6_H_10_O | 1230.8 | 503.169 | 1.56 ± 0.07^a^ | 1.15 ± 0.06^b^ | 1.48 ± 0.04^a^ | green, banana, fat |
| 22 | 1-octanal-D | C124130 | C_8_H_16_O | 1309 | 630.356 | 0.58 ± 0.05^a^ | 0.00 ± 0.00^b^ | 0.53 ± 0.01^a^ | aldehyde, waxy, citrus, orange, fruity, fatty |
| 23 | 1-octanal-M | C124130 | C_8_H_16_O | 1309.3 | 630.836 | 1.39 ± 0.04^a^ | 0.74 ± 0.01^b^ | 1.40 ± 0.01^a^ | aldehyde, waxy, citrus, orange, fruity, fatty |
| 24 | (E)-2-Heptenal-D | C18829555 | C_7_H_12_O | 1342.2 | 680.307 | 0.87 ± 0.04^a^ | 0.00 ± 0.00^c^ | 0.79 ± 0.02^b^ | spicy, green vegetables, fresh, fatty |
| 25 | (E)-2-Heptenal-M | C18829555 | C_7_H_12_O | 1343 | 681.649 | 1.11 ± 0.01^a^ | 0.58 ± 0.03^b^ | 1.17 ± 0.02^a^ | spicy, green vegetables, fresh, fatty |
| 26 | 1-nonanal-M | C124196 | C_9_H_18_O | 1401.1 | 779.078 | 1.02 ± 0.06^a^ | 0.64 ± 0.01^b^ | 1.09 ± 0.03^a^ | rose, citrus, strong oily |
| 27 | 1-nonanal-D | C124196 | C_9_H_18_O | 1401.5 | 779.959 | 0.38 ± 0.04^a^ | 0.00 ± 0.00^b^ | 0.42 ± 0.01^a^ | rose, citrus, strong oily |
| 28 | (E)-2-octenal | C2548870 | C_8_H_14_O | 1426.2 | 825.994 | 0.54 ± 0.02^b^ | 0.60 ± 0.03^a^ | 0.57 ± 0.01^ab^ | fresh cucumber, fatty, green herbal, banana, green leaf |
| 29 | 2-furaldehyde | C98011 | C_5_H_4_O_2_ | 1466.1 | 906.214 | 0.38 ± 0.02^b^ | 0.44 ± 0.02^a^ | 0.31 ± 0.01^c^ | sweet, woody, almond, bready |
| 30 | (E, E)-2,4-heptadienal | C4313035 | C_7_H_10_O | 1482.1 | 940.609 | 0.30 ± 0.03^a^ | 0.22 ± 0.03^b^ | 0.35 ± 0.02^a^ | fatty, oily, aldehyde, vegetable, cinnamon |
| 31 | Decanal | C112312 | C_10_H_20_O | 1489 | 955.649 | 0.22 ± 0.01^a^ | 0.00 ± 0.00^b^ | 0.25 ± 0.02^a^ | sweet, waxy, floral, citrus, aldehyde, fatty |
| 32 | Benzaldehyde-M | C100527 | C_7_H_6_O | 1501.3 | 983.51 | 1.84 ± 0.08^b^ | 1.83 ± 0.01^b^ | 2.16 ± 0.05^a^ | bitter almond, cherry, nutty |
| 33 | Benzaldehyde-D | C100527 | C_7_H_6_O | 1504.6 | 990.966 | 0.45 ± 0.05^b^ | 0.25 ± 0.02^c^ | 0.63 ± 0.05^a^ | bitter almond, cherry, nutty |
| 34 | (E)-4-Decenal | C65405701 | C_10_H_18_O | 1558.6 | 1123.565 | 0.18 ± 0.01^b^ | 0.48 ± 0.05^a^ | 0.20 ± 0.01^b^ | citrus |
| 35 | phenylacetaldehyde | C122781 | C_8_H_8_O | 1624.5 | 1309.402 | 1.36 ± 0.11^b^ | 0.83 ± 0.10^c^ | 1.86 ± 0.01^a^ | hyacinth, sweet fruity, almond, cherry, clover honey, cocoa |
| Alcohols | |  |  |  |  |  |  |  |  |
| 36 | Ethanol | C64175 | C_2_H_6_O | 953.5 | 206.163 | 1.25 ± 0.12^b^ | 0.98 ± 0.05^c^ | 1.51 ± 0.03^a^ | aromaticity |
| 37 | 2-butanol | C78922 | C_4_H_10_O | 1033 | 262.076 | 0.21 ± 0.01^a^ | 0.14 ± 0.01^b^ | 0.22 ± 0.01^a^ | fruity |
| 38 | 1-Propanol | C71238 | C_3_H_8_O | 1052.7 | 278.348 | 0.58 ± 0.05^a^ | 0.31 ± 0.01^c^ | 0.49 ± 0.00^b^ | alcohol, pungent |
| 39 | 1-Propanol, 2-methyl | C78831 | C_4_H_10_O | 1102 | 326.176 | 0.30 ± 0.04^ab^ | 0.39 ± 0.05^a^ | 0.20 ± 0.06^b^ | fresh, alcoholic, leather |
| 40 | 3-Pentanol | C584021 | C_5_H_12_O | 1116.3 | 343.283 | 0.33 ± 0.05^b^ | 0.00 ± 0.00^c^ | 0.40 ± 0.01^a^ |  |
| 41 | 2-Pentanol | C6032297 | C_5_H_12_O | 1128.1 | 358.139 | 0.23 ± 0.01^a^ | 0.00 ± 0.00^c^ | 0.18 ± 0.01^b^ | fusel oil, green |
| 42 | Butanol-M | C71363 | C_4_H_10_O | 1150.3 | 387.669 | 0.54 ± 0.01^a^ | 0.31 ± 0.02^b^ | 0.58 ± 0.02^a^ | wine |
| 43 | Butanol-D | C71363 | C_4_H_10_O | 1152.4 | 390.604 | 0.09 ± 0.01^a^ | 0.00 ± 0.00^b^ | 0.09 ± 0.01^a^ | wine |
| 44 | 1,4-Cineol-D | C470677 | C_10_H_18_O | 1207.8 | 469.295 | 0.28 ± 0.02^b^ | 0.71 ± 0.05^a^ | 0.33 ± 0.02^b^ | cool, camphor, spice |
| 45 | 1,4-Cineol-M | C470677 | C_10_H_18_O | 1208.3 | 470.063 | 0.48 ± 0.03^c^ | 0.97 ± 0.01^a^ | 0.60 ± 0.04^b^ | cool, camphor, spice |
| 46 | 2-methyl-1-butanol | C137326 | C_5_H_12_O | 1212.2 | 475.688 | 0.15 ± 0.01^b^ | 0.17 ± 0.01^ab^ | 0.19 ± 0.02^a^ | roast onion, fruity, floral, wine |
| 47 | 1-Butanol, 3-methyl-M | C123513 | C_5_H_12_O | 1213.9 | 478.093 | 0.94 ± 0.02^a^ | 0.75 ± 0.03^b^ | 1.01 ± 0.04^a^ | whiskey, banana, fruity |
| 48 | 1-Butanol, 3-methyl-D | C123513 | C_5_H_12_O | 1214.6 | 479.124 | 0.88 ± 0.04^b^ | 0.23 ± 0.02^c^ | 0.96 ± 0.02^a^ | whiskey, banana, fruity |
| 49 | 1-Pentanol-M | C71410 | C_5_H_12_O | 1263.6 | 555.567 | 0.69 ± 0.04^b^ | 0.49 ± 0.03^c^ | 0.87 ± 0.03^a^ | balsamic |
| 50 | 1-Pentanol-D | C71410 | C_5_H_12_O | 1264.9 | 557.804 | 0.19 ± 0.02^b^ | 0.00 ± 0.00^c^ | 0.25 ± 0.01^a^ | balsamic |
| 51 | 1-pentanol-4-methyl | C626891 | C_6_H_14_O | 1336.2 | 671.102 | 0.17 ± 0.02^b^ | 0.16 ± 0.00^b^ | 0.22 ± 0.00^a^ | nutty |
| 52 | (Z)-2-Penten-1-ol | C1576950 | C_5_H_10_O | 1345.7 | 685.914 | 0.42 ± 0.07^a^ | 0.21 ± 0.01^b^ | 0.50 ± 0.01^a^ | green, plastic, rubber |
| 53 | 1-Hexanol-M | C111273 | C_6_H_14_O | 1370.8 | 726.564 | 0.63 ± 0.05^a^ | 0.47 ± 0.01^b^ | 0.66 ± 0.01^a^ | fresh, fruity, wine, sweet, green |
| 54 | 1-Hexanol-D | C111273 | C_6_H_14_O | 1374 | 731.866 | 0.11 ± 0.02^a^ | 0.00 ± 0.00^b^ | 0.10 ± 0.01^a^ | fresh, fruity, wine, sweet, green |
| 55 | (Z)-3-hexen-1-ol | C928961 | C_6_H_12_O | 1397.3 | 772.23 | 0.18 ± 0.00^a^ | 0.18 ± 0.02^a^ | 0.17 ± 0.01^a^ | green, herb |
| 56 | 1-Octen-3-ol-M | C3391864 | C_8_H_16_O | 1458.2 | 889.643 | 0.24 ± 0.00^b^ | 2.12 ± 0.09^a^ | 0.28 ± 0.06^b^ | mushroom, lavender, rose, hay |
| 57 | 1-Octen-3-ol-D | C3391864 | C_8_H_16_O | 1459.4 | 892.133 | 0.00 ± 0.00^b^ | 0.48 ± 0.08^a^ | 0.00 ± 0.00^b^ | mushroom, lavender, rose, hay |
| 58 | 2-ethylhexanol | C104767 | C_8_H_18_O | 1496.1 | 971.706 | 0.43 ± 0.01^a^ | 0.24 ± 0.04^b^ | 0.41 ± 0.02^a^ | citrus, fresh floral, greasy |
| 59 | 1,2-ethanediol | C107211 | C_2_H_6_O_2_ | 1649 | 1386.116 | 0.67 ± 0.03^b^ | 0.88 ± 0.10^a^ | 0.58 ± 0.03^b^ | odorless |
| Ketones | |  |  |  |  |  |  |  |  |
| 60 | 2-propanone | C67641 | C_3_H_6_O | 836.9 | 155.686 | 6.77 ± 0.09^b^ | 8.50 ± 0.18^a^ | 6.89 ± 0.15^b^ | fresh, apple, pear |
| 61 | 2-Butanone | C78933 | C_4_H_8_O | 926.8 | 190.557 | 1.47 ± 0.25^b^ | 4.57 ± 0.19^a^ | 1.08 ± 0.15^b^ | fruity, camphor |
| 62 | 2,3-Butanedione | C431038 | C_4_H_6_O_2_ | 988.4 | 228.606 | 1.31 ± 0.04^b^ | 1.17 ± 0.05^c^ | 1.43 ± 0.02^a^ | butter, popcorn, sweet taste, sour rice |
| 63 | 2-Pentanone | C107879 | C_5_H_10_O | 995.3 | 233.499 | 0.21 ± 0.02^b^ | 0.50 ± 0.03^a^ | 0.16 ± 0.01^b^ | acetone, fresh, sweet fruity, wine |
| 64 | 1-Penten-3-one-D | C1629589 | C_5_H_8_O | 1038.2 | 266.285 | 1.27 ± 0.02^a^ | 0.48 ± 0.04^b^ | 1.31 ± 0.03^a^ | strong pungent odors |
| 65 | 1-Penten-3-one-M | C1629589 | C_5_H_8_O | 1040.6 | 268.19 | 0.71 ± 0.02^a^ | 0.63 ± 0.03^b^ | 0.72 ± 0.01^a^ | strong pungent odors |
| 66 | 3-Penten-2-one | C625332 | C_5_H_8_O | 1133.3 | 364.772 | 0.36 ± 0.03^b^ | 0.54 ± 0.04^a^ | 0.40 ± 0.02^b^ | fruity, turns into spicy during storage |
| 67 | 4-Heptanone | C123193 | C_7_H_14_O | 1159.4 | 400.537 | 0.57 ± 0.03^b^ | 0.92 ± 0.05^a^ | 0.60 ± 0.02^b^ | fruity |
| 68 | 2-Heptanone | C110430 | C_7_H_14_O | 1188.1 | 442.194 | 0.00 ± 0.00^b^ | 0.09 ± 0.01^a^ | 0.00 ± 0.00^b^ | pear, banana, fruity, slight medicinal fragrance |
| 69 | 2-Octanone | C111137 | C_8_H_16_O | 1303.1 | 621.931 | 0.31 ± 0.05^a^ | 0.28 ± 0.01^ab^ | 0.22 ± 0.01^b^ | mouldy, ketone, milk, cheese, mushroom |
| 70 | Cyclohexanone | C108941 | C_6_H_10_O | 1317.7 | 643.185 | 0.20 ± 0.01^a^ | 0.24 ± 0.01^a^ | 0.20 ± 0.02^a^ | strong pungent, earthy |
| 71 | 1-Octen-3-one-M | C4312996 | C_8_H_14_O | 1321.3 | 648.522 | 0.65 ± 0.03^a^ | 0.49 ± 0.03^b^ | 0.62 ± 0.02^a^ | strong earthy, mushroom, vegetable, fishy, chicken |
| 72 | 1-Octen-3-one-D | C4312996 | C_8_H_14_O | 1321.6 | 648.933 | 0.23 ± 0.01^a^ | 0.00 ± 0.00^c^ | 0.16 ± 0.01^b^ | strong earthy, mushroom, vegetable, fishy, chicken |
| 73 | 2,2,6-trimethylcyclohexanone | C2408379 | C_9_H_16_O | 1329.3 | 660.525 | 0.39 ± 0.02^a^ | 0.38 ± 0.02^a^ | 0.39 ± 0.01^a^ | camphor, thujonic |
| 74 | 5-Nonanone | C502567 | C_9_H_18_O | 1345 | 684.727 | 0.16 ± 0.01^b^ | 0.31 ± 0.02^a^ | 0.14 ± 0.01^b^ | special aromatic aroma |
| 75 | 2-methyl-2-hepten-6-one | C110930 | C_8_H_14_O | 1354.5 | 699.927 | 4.00 ± 0.14^b^ | 3.97 ± 0.08^b^ | 4.51 ± 0.10^a^ | citrus, fruity, mouldy, ketone |
| 76 | 2-Methyl-2-cyclopenten-1-one | C1120736 | C_6_H_8_O | 1384.6 | 750.043 | 0.12 ± 0.01^a^ | 0.00 ± 0.00^b^ | 0.12 ± 0.01^a^ | fresh, apple, pear |
| Esters | |  |  |  |  |  |  |  |  |
| 77 | Acetic acid ethyl ester | C141786 | C_4_H_8_O_2_ | 910.2 | 182.433 | 4.19 ± 0.52^a^ | 1.02 ± 0.31^b^ | 4.67 ± 0.46^a^ | fresh, fruity, sweet, grassy |
| 78 | Acetic acid butyl ester | C123864 | C_6_H_12_O_2_ | 1082.6 | 305.017 | 0.10 ± 0.01^a^ | 0.00 ± 0.00^b^ | 0.10 ± 0.00^a^ | fruity |
| 79 | Ethyl formate | C109944 | C_3_H_6_O_2_ | 816.7 | 149.016 | 0.36 ± 0.02^c^ | 0.80 ± 0.02^a^ | 0.52 ± 0.02^b^ | spicy and stimulating taste, pineapple, rum |
| 80 | 1-Butanol, 3-methyl-, acetate | C123922 | C_7_H_14_O_2_ | 1127.1 | 356.843 | 0.08 ± 0.01^a^ | 0.00 ± 0.00^b^ | 0.08 ± 0.01^a^ | sweet, banana, fruity |
| 81 | 2-Methylbutyl acetate | C624419 | C_7_H_14_O_2_ | 1146.6 | 382.657 | 0.10 ± 0.00^a^ | 0.00 ± 0.00^b^ | 0.11 ± 0.01^a^ | fruity |
| 82 | beta-Phenylethyl acetate | C103457 | C_10_H_12_O_2_ | 1886 | 2404.731 | 0.96 ± 0.09^ab^ | 1.20 ± 0.20^a^ | 0.85 ± 0.07^b^ | sweet, rose, honey, apple, cocoa |
| Acids | |  |  |  |  |  |  |  |  |
| 83 | Acetic acid-D | C64197 | C_2_H_4_O_2_ | 1462.9 | 899.527 | 0.78 ± 0.02^a^ | 0.61 ± 0.02^a^ | 0.63 ± 0.20^a^ | spicy |
| 84 | Acetic acid-M | C64197 | C_2_H_4_O_2_ | 1466.5 | 907.091 | 2.02 ± 0.06^a^ | 2.11 ± 0.09^a^ | 1.97 ± 0.23^a^ | spicy |
| 85 | Propanoic acid | C79094 | C_3_H_6_O_2_ | 1546.4 | 1092.198 | 0.51 ± 0.12^a^ | 0.47 ± 0.02^a^ | 0.33 ± 0.07^a^ | yogurt, vinegar |
| Terpenes | |  |  |  |  |  |  |  |  |
| 86 | alpha-Terpinene | C99865 | C_10_H_16_ | 1180 | 431.141 | 0.16 ± 0.01^a^ | 0.16 ± 0.02^a^ | 0.14 ± 0.01^a^ | woody, lemon, citrus |
| 87 | (Z)-Ocimene-M | C470826 | C_10_H_18_O | 1234.5 | 508.86 | 0.46 ± 0.02^b^ | 0.95 ± 0.04^a^ | 0.50 ± 0.00^b^ | camphor, refreshing herbal |
| 88 | (Z)-Ocimene-D | C470826 | C_10_H_18_O | 1235 | 509.629 | 0.28 ± 0.02^b^ | 0.45 ± 0.05^a^ | 0.27 ± 0.01^b^ | camphor, refreshing herbal |
| 89 | (R/S)-linalool | C78706 | C_10_H_18_O | 1554.3 | 1112.266 | 0.31 ± 0.01^a^ | 0.28 ± 0.07^a^ | 0.39 ± 0.03^a^ | citrus, rose, woody, blueberry |
| 90 | isomenthone | C491076 | C_10_H_18_O | 1514.6 | 1014.373 | 0.09 ± 0.01^a^ | 0.00 ± 0.00^b^ | 0.10 ± 0.01^a^ | mint, cool |
| 91 | delta-Cadinene | C483761 | C_15_H_24_ | 1770.2 | 1837.312 | 6.77 ± 0.66^a^ | 7.65 ± 0.26^a^ | 6.57 ± 0.45^a^ | thyme, herbal, woody |
| 92 | (E)-beta-Damascenone | C23726934 | C_13_H_18_O | 1884.4 | 2395.67 | 1.14 ± 0.06^a^ | 0.00 ± 0.00^c^ | 0.91 ± 0.06^b^ | apple, fruity, rose, honey, tobacco, sweet |
| Nitrogen compounds | |  |  |  |  |  |  |  |  |
| 93 | Pyridine | C110861 | C_5_H_5_N | 1203.6 | 463.468 | 0.23 ± 0.03^a^ | 0.00 ± 0.00^c^ | 0.18 ± 0.01^b^ | nut, musty |
| 94 | Pyrazine | C290379 | C_4_H_4_N_2_ | 1223.6 | 492.38 | 0.08 ± 0.01^b^ | 0.12 ± 0.01^a^ | 0.08 ± 0.01^b^ | nutty, mouldy, roast, earthy |
| 95 | 2-Methylpyrazine-D | C109080 | C_5_H_6_N_2_ | 1281.9 | 587.286 | 0.00 ± 0.00^b^ | 0.09 ± 0.00^a^ | 0.00 ± 0.00^b^ | nutty, mouldy, roast, earthy |
| 96 | 2-Methylpyrazine-M | C109080 | C_5_H_6_N_2_ | 1282 | 587.382 | 0.11 ± 0.01^b^ | 0.80 ± 0.03^a^ | 0.15 ± 0.03^b^ | grass |
| 97 | 2-Ethylpyridine | C100710 | C_7_H_9_N | 1296.3 | 612.206 | 0.00 ± 0.00^b^ | 0.23 ± 0.01^a^ | 0.00 ± 0.00^b^ | nutty, peanut, mouldy, earthy, potato, fatty, cocoa powder |
| 98 | 2,5-Dimethylpyrazine | C123320 | C_6_H_8_N_2_ | 1338.9 | 675.283 | 0.00 ± 0.00^b^ | 0.14 ± 0.00^a^ | 0.00 ± 0.00^b^ | roast, coffee, peanut, potato |
| 99 | 2,6-dimethylpyrazine | C108509 | C_6_H_8_N_2_ | 1343.7 | 682.787 | 0.00 ± 0.00^b^ | 0.34 ± 0.01^a^ | 0.00 ± 0.00^b^ | nutty |
| 100 | 2,3-Dimethylpyrazine | C5910894 | C_6_H_8_N_2_ | 1360.3 | 709.192 | 0.00 ± 0.00^b^ | 0.13 ± 0.01^a^ | 0.00 ± 0.00^b^ | nutty, peanut, bready |
| 101 | 2-Ethyl-3-methylpyrazine | C15707230 | C_7_H_10_N_2_ | 1403.4 | 783.251 | 0.09 ± 0.01^a^ | 0.00 ± 0.00^b^ | 0.09 ± 0.00^a^ | burnt, coffee, nutty, roasted woody |
| 102 | 2,6-Dimethyl-3-ethylpyrazine | C13925070 | C_8_H_12_N_2_ | 1472.7 | 920.229 | 0.15 ± 0.02^a^ | 0.00 ± 0.00^b^ | 0.17 ± 0.01^a^ | sweet, fruity, nuts |
| 103 | 1H-pyrrole | C109977 | C_4_H_5_N | 1513.8 | 1012.419 | 0.30 ± 0.01^b^ | 0.62 ± 0.01^a^ | 0.31 ± 0.01^b^ | foul |
| 104 | N,N-Dimethylformamide | C68122 | C_3_H_7_NO | 1349.9 | 692.51 | 0.29 ± 0.03^b^ | 0.48 ± 0.04^a^ | 0.18 ± 0.02^c^ | unpleasant odor |
|  | Sulfur compounds |  |  |  |  |  |  |  |  |
| 105 | Dimethyl sulfide | C75183 | C_2_H_6_S | 777.4 | 136.878 | 0.35 ± 0.04^b^ | 0.48 ± 0.02^a^ | 0.40 ± 0.01^b^ | cabbage, sulfur, gasoline |
| 106 | diethyl disulfide | C110816 | C_4_H_10_S_2_ | 1230.8 | 503.099 | 0.21 ± 0.01^b^ | 0.28 ± 0.02^a^ | 0.18 ± 0.01^b^ | pungent, pong |
| 107 | methyl thiocyanate | C556649 | C_2_H_3_NS | 1289 | 600.046 | 0.11 ± 0.01^b^ | 0.00 ± 0.00^c^ | 0.15 ± 0.01^a^ | garlic, onion, sulfur |
| 108 | 2-furanmethanethiol | C98022 | C_5_H_6_OS | 1449.1 | 871.099 | 0.06 ± 0.00^b^ | 0.00 ± 0.00^c^ | 0.12 ± 0.02^a^ | sulfury, coffee, fat, smoky |
| 109 | 2-acetyl-2-thiazoline | C29926418 | C_5_H_7_NOS | 1783.2 | 1893.74 | 0.00 ± 0.00^b^ | 1.29 ± 0.09^a^ | 0.00 ± 0.00^b^ | roast, popcorn |
|  | Oxygenated compounds |  |  |  |  |  |  |  |  |
| 110 | 1-Hydroxy-2-propanone | C116096 | C_3_H_6_O_2_ | 1324.5 | 653.302 | 0.09 ± 0.01^b^ | 0.18 ± 0.03^a^ | 0.08 ± 0.01^b^ | pungent, caramel, fresh |
| Furans | |  |  |  |  |  |  |  |  |
| 111 | Tetrahydrofuran | C109999 | C_4_H_8_O | 897.4 | 177.431 | 0.79 ± 0.13^b^ | 1.69 ± 0.30^a^ | 0.70 ± 0.11^b^ | ether |
| 112 | 2-pentylfuran | C3777693 | C_9_H_14_O | 1239.7 | 516.943 | 0.47 ± 0.03^a^ | 0.47 ± 0.03^a^ | 0.37 ± 0.05^b^ | bean, fruity, earthy, green, vegetable |
| Aromatics | |  |  |  |  |  |  |  |  |
| 113 | 1,2-dimethylbenzene | C95476 | C_8_H_10_ | 1190.9 | 445.902 | 0.11 ± 0.00^a^ | 0.00 ± 0.00^b^ | 0.11 ± 0.01^a^ | geranium |
| Others | |  |  |  |  |  |  |  |  |
| 114 | 27 |  |  | 733.3 | 124.428 | 0.87 ± 0.04^b^ | 1.41 ± 0.06^a^ | 0.83 ± 0.01^b^ |  |
| 115 | 7 |  |  | 949.9 | 204.002 | 0.25 ± 0.03^b^ | 0.47 ± 0.02^a^ | 0.18 ± 0.02^c^ |  |
| 116 | 1 |  |  | 968.8 | 215.711 | 1.22 ± 0.05^a^ | 0.61 ± 0.03^c^ | 1.08 ± 0.04^b^ |  |
| 117 | 29 |  |  | 1008.1 | 242.844 | 0.15 ± 0.05^a^ | 0.00 ± 0.00^b^ | 0.18 ± 0.01^a^ |  |
| 118 | 28 |  |  | 1014.1 | 247.312 | 0.22 ± 0.02^b^ | 0.42 ± 0.03^a^ | 0.20 ± 0.00^b^ |  |
| 119 | 25 |  |  | 1022.8 | 253.956 | 0.00 ± 0.00^b^ | 0.32 ± 0.04^a^ | 0.00 ± 0.00^b^ |  |
| 120 | 3 |  |  | 1028.7 | 258.663 | 0.31 ± 0.06^a^ | 0.21 ± 0.01^b^ | 0.25 ± 0.02^ab^ |  |
| 121 | 30 |  |  | 1031.3 | 260.717 | 0.00 ± 0.00^b^ | 0.31 ± 0.02^a^ | 0.00 ± 0.00^b^ |  |
| 122 | 2 |  |  | 1050.3 | 276.311 | 0.44 ± 0.03^a^ | 0.49 ± 0.01^a^ | 0.47 ± 0.03^a^ |  |
| 123 | 5 |  |  | 1078.5 | 301.227 | 0.61 ± 0.05^a^ | 0.29 ± 0.02^b^ | 0.59 ± 0.01^a^ |  |
| 124 | 41 |  |  | 1086.8 | 309.042 | 0.00 ± 0.00^b^ | 0.21 ± 0.07^a^ | 0.00 ± 0.00^b^ |  |
| 125 | 6 |  |  | 1097.3 | 320.731 | 0.22 ± 0.03^a^ | 0.13 ± 0.02^b^ | 0.19 ± 0.01^a^ |  |
| 126 | 31 |  |  | 1102.2 | 326.395 | 0.00 ± 0.00^b^ | 0.45 ± 0.07^a^ | 0.00 ± 0.00^b^ |  |
| 127 | 8 |  |  | 1135.9 | 368.166 | 0.00 ± 0.00^b^ | 0.39 ± 0.19^a^ | 0.00 ± 0.00^b^ |  |
| 128 | 9 |  |  | 1160 | 401.391 | 0.71 ± 0.03^b^ | 0.93 ± 0.03^a^ | 0.49 ± 0.03^c^ |  |
| 129 | 4 |  |  | 1190.9 | 445.971 | 0.00 ± 0.00^b^ | 0.11 ± 0.01^a^ | 0.00 ± 0.00^b^ |  |
| 130 | 13 |  |  | 1210.5 | 473.137 | 0.35 ± 0.03^a^ | 0.38 ± 0.01^a^ | 0.36 ± 0.02^a^ |  |
| 131 | 14 |  |  | 1215.3 | 480.051 | 0.00 ± 0.00^b^ | 0.66 ± 0.05^a^ | 0.00 ± 0.00^b^ |  |
| 132 | 15 |  |  | 1232.8 | 506.172 | 0.14 ± 0.02^a^ | 0.00 ± 0.00^b^ | 0.16 ± 0.01^a^ |  |
| 133 | 10 |  |  | 1247.7 | 529.603 | 0.45 ± 0.02^b^ | 0.53 ± 0.01^a^ | 0.38 ± 0.01^c^ |  |
| 134 | 12 |  |  | 1254.7 | 540.832 | 0.50 ± 0.03^a^ | 0.39 ± 0.01^b^ | 0.45 ± 0.02^a^ |  |
| 135 | 11 |  |  | 1262.7 | 554.101 | 4.62 ± 0.17^a^ | 3.54 ± 0.16^b^ | 3.77 ± 0.08^b^ |  |
| 136 | 16 |  |  | 1267.6 | 562.451 | 0.00 ± 0.00^b^ | 0.40 ± 0.04^a^ | 0.00 ± 0.00^b^ |  |
| 137 | 23 |  |  | 1276.3 | 577.474 | 0.14 ± 0.00^a^ | 0.00 ± 0.00^b^ | 0.14 ± 0.01^a^ |  |
| 138 | 34 |  |  | 1281.4 | 586.472 | 0.12 ± 0.00^b^ | 0.65 ± 0.01^a^ | 0.12 ± 0.01^b^ |  |
| 139 | 32 |  |  | 1288.6 | 599.29 | 0.07 ± 0.00^b^ | 0.00 ± 0.00^c^ | 0.10 ± 0.01^a^ |  |
| 140 | 19 |  |  | 1309.3 | 630.868 | 0.07 ± 0.00^b^ | 0.12 ± 0.02^a^ | 0.07 ± 0.01^b^ |  |
| 141 | 17 |  |  | 1317.8 | 643.325 | 0.10 ± 0.01^b^ | 1.04 ± 0.01^a^ | 0.11 ± 0.02^b^ |  |
| 142 | 18 |  |  | 1335.4 | 669.871 | 0.83 ± 0.01^a^ | 0.30 ± 0.02^c^ | 0.66 ± 0.03^b^ |  |
| 143 | 35 |  |  | 1350.2 | 693.071 | 0.48 ± 0.05^b^ | 0.25 ± 0.01^c^ | 0.65 ± 0.01^a^ |  |
| 144 | 26 |  |  | 1359.3 | 707.658 | 0.00 ± 0.00^b^ | 0.08 ± 0.01^a^ | 0.00 ± 0.00^b^ |  |
| 145 | 22 |  |  | 1361 | 710.358 | 0.00 ± 0.00^b^ | 0.90 ± 0.05^a^ | 0.00 ± 0.00^b^ |  |
| 146 | 38 |  |  | 1363.2 | 713.926 | 0.42 ± 0.02^a^ | 0.26 ± 0.00^b^ | 0.41 ± 0.02^a^ |  |
| 147 | 21 |  |  | 1369 | 723.586 | 0.00 ± 0.00^b^ | 0.48 ± 0.02^a^ | 0.00 ± 0.00^b^ |  |
| 148 | 33 |  |  | 1375.6 | 734.613 | 0.00 ± 0.00^b^ | 0.23 ± 0.02^a^ | 0.00 ± 0.00^b^ |  |
| 149 | 37 |  |  | 1383.7 | 748.505 | 0.16 ± 0.00^b^ | 0.00 ± 0.00^c^ | 0.19 ± 0.01^a^ |  |
| 150 | 20 |  |  | 1406.2 | 788.444 | 1.14 ± 0.03^b^ | 1.10 ± 0.01^b^ | 1.22 ± 0.03^a^ |  |
| 151 | 39 |  |  | 1407.6 | 791.064 | 1.11 ± 0.02^b^ | 0.56 ± 0.03^c^ | 1.22 ± 0.03^a^ |  |
| 152 | 40 |  |  | 1417.7 | 809.806 | 0.22 ± 0.04^a^ | 0.00 ± 0.00^b^ | 0.17 ± 0.01^a^ |  |
| 153 | 24 |  |  | 1439.6 | 852.023 | 0.16 ± 0.02^a^ | 0.16 ± 0.03^a^ | 0.20 ± 0.00^a^ |  |
| 154 | 36 |  |  | 1440.6 | 854.022 | 0.22 ± 0.01^b^ | 0.36 ± 0.01^a^ | 0.19 ± 0.01^c^ |  |

**Note:** Values are presented as the mean ± SD of three replicate samples. Different lowercase superscript letters within the same row indicate significant differences among the normal leaves, *Penicillium*, and *Penicillium*+ZD-F13 groups based on one-way ANOVA followed by Tukey’s HSD test (*P* < 0.05). Groups sharing at least one letter are not significantly different.

**Figure S1. Antifungal activity and extracellular metabolite analysis of *B. velezensis* ZD-F13.** (A) Antifungal activity of the ZD-F13 bacterial suspension against *P. chrysogenum* Z1. (B) Antifungal activity of the methanolic extract from the ZD-F13 fermentation supernatant. (C) HPLC chromatograms of the iturin A standard and the methanolic extract from the ZD-F13 fermentation supernatant.

**Figure S2. GC-IMS fingerprint** **profiles of VOCs across different groups.** The fingerprint plot was divided into two panels (A and B) to improve readability. Red boxes labeled a-d indicate representative VOC regions showing distinct differences among the normal leaves, *Penicillium*, and *Penicillium*+ZD-F13 groups.

**Figure S3. VOCs with VIP > 1 identified by OPLS-DA.** These VOCs were considered important variables contributing to group separation.

**Figure S4. Differential** **abundance analyses of bacterial taxa between the groups.** (A) Wilcoxon rank-sum test for differential taxa. (B) LEfSe analysis identifying taxa with significant differences.

**Figure S5. Differential abundance analyses of fungal taxa between the groups.** (A) Wilcoxon rank-sum test for differential taxa. (B) LEfSe analysis identifying taxa with significant differences.
